# Five years follow up of patient receiving prolonged mechanical ventilation: Data for a single center in Taiwan

**DOI:** 10.3389/fmed.2022.1038915

**Published:** 2022-11-18

**Authors:** Chienhsiu Huang

**Affiliations:** Department of Internal Medicine, Division of Chest Medicine, Dalin Tzu Chi Hospital, Buddhist Tzu Chi Medical Foundation, Chiayi, Taiwan

**Keywords:** prolonged mechanical ventilation, respiratory care center, 5-year mortality rate, weaning unit, successful weaning

## Abstract

**Background:**

The National Association for Medical Direction of Respiratory Care recommended tracking 1-year survival rates (the most relevant outcome) in patients treated with prolonged mechanical ventilation. However, patients treated with prolonged mechanical ventilation had higher mortality rates within the first 2 years after weaning. More knowledge regarding long-term mortality would help patients, families, and clinicians choose appropriate interventions and make end-of-life decisions. In this investigation, we attempted to determine the rates of long-term mortality for all patients treated with prolonged mechanical ventilation over a period of 10 years.

**Objective:**

The purpose of this investigation was to enhance the overall survival outcomes for patients receiving prolonged mechanical ventilation by identifying the factors affecting the 5-year mortality rates for these patients.

**Design:**

Retrospective observational study.

**Materials and methods:**

In this retrospective study, we explored the influential factors related to the overall survival outcomes of all patients treated with prolonged mechanical ventilation. We enrolled every individual admitted to the weaning unit between January 1, 2012, and December 31, 2016. The length of survival for each patient was estimated from admission to the weaning unit until death or December 31, 2021, whichever came first. We analyzed the data to investigate the survival time, mortality rates, and survival curves in these patients.

**Results:**

Long-term follow-up information was gathered for 296 patients who received prolonged mechanical ventilation. There was better mean survival times in patients treated with prolonged mechanical ventilation with the following characteristics (in order): no comorbidities, tracheostomies, and intracranial hemorrhage. Successful weaning, receipt of tracheostomy, an age less than 75 years, and no comorbidities were associated with better long-term overall survival outcomes.

**Conclusion:**

Prolonged mechanical ventilation patients had abysmal overall survival outcomes. Even though prolonged mechanical ventilation patients’ long-term survival outcomes are tragic, medical professionals should never give up on the dream of enhancing long-term outcomes.

## Background

A conference on the treatment and management of prolonged mechanical ventilation (PMV) patients was held in 2004 by the National Association for Medical Direction of Respiratory Care. The most relevant outcome, according to consensus, is the 1-year survival rate (1). Many PMV patients were discharged from the hospital but were again readmitted after a year. Some patients needed long-term ventilator support for over a year. According to the study conducted by Stoller, patients who were discharged from the weaning unit had a mortality rate that fell by 68% within the first 2 years and then fell slower after that (2). In accordance with Aboussouan’s study, 40% of PMV patients were still living by the second and third years after discharge (3). The clinical course of PMV patients continues to progress after 1 year. The medical profession pays little attention to the long-term survival rates of PMV patients, and there is less information available on these patients’ long-term mortality. The evaluation of the long-term outcome of patients undergoing PMV should take more than 1 year.

The comprehensive care program for ventilator-dependent patients encompasses mechanical ventilator care in four settings: the intensive care unit (also known as the acute critical care stage), the respiratory care center (also known as the RCC), the respiratory care ward (also known as the RCW), and home care services (a stable period in which the patient is cared for directly by family caregivers or nurses who work in nursing homes) in Taiwan (4).

In this study, throughout a 10-year follow-up period, we aimed to enhance the overall survival outcomes for patients receiving prolonged mechanical ventilation. We investigate the long-term mortality of all PMV patients at a single weaning unit in the acute care hospital by identifying the factors affecting the 5-year mortality rates for these patients. Our study on long-term mortality is significant because it advances our clinical knowledge of patients receiving PMV, which is useful for making decisions about their long-term care and end-of-life options.

## Materials and methods

### Study design

In this retrospective study, we explored the influential factors related to the overall survival outcomes of all PMV patients. We enrolled every individual admitted to the weaning unit between January 1, 2012, and December 31, 2016. The length of survival for each patient was estimated from admission to the weaning unit until death or December 31, 2021, whichever came first. This means that every patient has been followed up for at least 5 years and confirmed mortality data are available for a minimum of 5 years. We documented their age, sex, comorbidities, weaning status, receipt or non-receipt of tracheostomy, causes of respiratory failure led to PMV, survival time, mortality rates, and long-term survival outcomes. Retrospective data collection from the patients’ medical records was performed. We compared clinical variables and receipt or non-receipt of tracheostomy between PMV patients who survived < 5 years and those who survived ≥ 5 years. In addition, six survival curves were generated for all PMV patients, including the following: (1) Successful weaned PMV patients versus unsuccessful weaned PMV patients; (2) Receipt or non-receipt of tracheostomy PMV patients; (3) Aged < 75 years versus aged ≥ 75 years patients; (4) All PMV patients among the causes of respiratory failure led to PMV; (5) All PMV patients among different medical comorbidities; (6) All PMV patients among the number of comorbidities.

### Definitions and outcomes

We recently published six PMV articles in the literature. The hospital details, patient details, comorbidities, causes of acute respiratory failure leading to PMV, and eligibility criteria for RCC admission were the same as those in previous studies (5).

Causes of death in PMV patients:

All patients who had PMV had their causes of death compiled. Our PMV patients had seven major causes of mortality recorded, including (1) pneumonia, as defined by the Infectious Diseases Society of America (6); and (2) sepsis, with an infection etiology other than pneumonia; (3) respiratory failure, wherein the patient did not acquire pneumonia but experienced sputum impaction, respiratory distress, and hypoxemia; (4) sudden death, wherein the patient experienced a sudden onset of apnea and asystole; (5) cardiogenic shock, wherein the patient died due to underlying heart disease; (6) malignant disease, wherein the patient died due to underlying malignant disease; and (7) chronic obstructive pulmonary disease (COPD). We then gathered the pneumonia pathogens from PMV patients whose cause of death was pneumonia.

### Statistical analysis

To assess differences in age, weaning status, tracheostomy receipt or non-receipt, causes of acute respiratory failure requiring PMV, medical comorbidities, and a number of comorbidities, Student’s *t* test for continuous variables and Pearson’s chi-square test and Fisher’s exact test for categorical variables were used. Student’s *t* test and one-way analysis of variance were performed to compare survival times. Using Fisher’s exact test, mortality rates were compared. Using logistic regression, the correlation of each variable with less than 5-year and more than 5-year survival rates in PMV patients was studied. The association of each variable between the two patient groups was examined using univariate analysis. In the univariate analysis, the factors had a *P* value less than 0.05 and were incorporated into the multivariate analysis to determine the impact of each variable on the two patient groups. In this study, six survival curves were generated using the Kaplan–Meier method to determine the cumulative likelihood of survival as a function of the number of months. The following six survival curves for the patient groups were: (1) successfully weaned patients versus unsuccessfully weaned patients, (2) receipt versus non-receipt tracheostomy patients, (3) patients aged < 75 years versus those aged ≥ 75 years, (4) PMV patients among the causes of respiratory failure led to PMV, (5) PMV patients among different medical comorbidities, and 6. PMV patients are among the number of comorbidities. Using the log-rank test, the survival rates of the six survival curves were compared. Using Cox proportional hazards models, the link between the survival rates of the six survival curves was determined.

## Results

**FIGURE 1:**
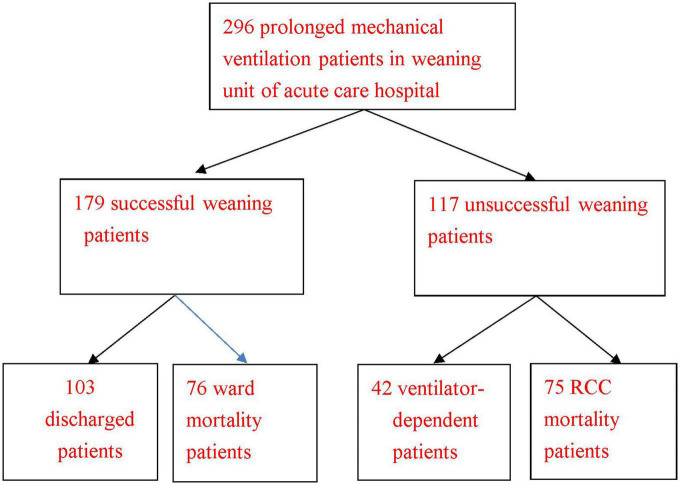
Clinical outcomes of 296 prolonged mechanical ventilation patients in the weaning unit of an acute care hospital. RCC: respiratory care center. Discharged prolonged mechanical ventilation patient was defined as a successfully weaned prolonged mechanical ventilation patient who was discharged from the hospital. Ward mortality prolonged mechanical ventilation patient was defined as a successfully weaned PMV patient who died in the hospital before discharge. RCC mortality prolonged mechanical ventilation patient was defined as a patient who died in the RCC.

We were able to collect long-term follow-up information on 296 PMV patients over the course of the 10-year research period. Of these, 189 (63.9%) were men, and 107 (36.1%) were women. There were 179 successful weaning PMV patients (including 103 discharged PMV patients and 76 ward mortality PMV patients), and 117 unsuccessful weaning PMV patients (including 42 ventilator-dependent PMV patients and 75 RCC mortality PMV patients (Figure 1). The mean age was 72.9 (range from 18 to 97 years). The mean survival time of 296 PMV patients is listed in Table 1. The better mean survival times in PMV patients were, in order, no comorbidities patients (1127.2 days), receipt of tracheostomy patients (783.3 days), and intracranial hemorrhage (ICH) patients (753.5 days). There were statistically significant differences in survival time in the following patient groups: (1) Successfully weaned patients and unsuccessfully weaned patients (*P* < 0.001); (2) Receipt or non-receipt of tracheostomy patients (*P* < 0.001); (3) Age < 75 years and ≥ 75 years patients (*P* < 0.001); (4) PMV patients among the causes of respiratory failure led to PMV (*P* = 0.045) (main related to ICH patients); (5) PMV patients among the number of comorbidities (*P* < 0.001). No differences in survival time were found among PMV patients with different medical comorbidities.

**TABLE 1:** The mean survival time of 296 prolonged mechanical ventilation patients.

Variable, (patient number)	Mean survival time (days)	P-value*
All patients (296)	417.9	**NA**
Successfully wean (179)	578.4	0.049
Unsuccessfully wean (117)	172.3	0.003
Tracheostomy (45)	783.3	0.008
Non-tracheostomy (251)	352.4	0.333
Ventilator-dependent (42)	451.8	0.612
**Age groups**		
Age <75 Y/O (131)	640.1	< 0.001
Age ≥75 Y/O (165)	241.4	0.015
**Causes of respiratory failure led to PMV**		
Pneumonia (104)	288.5	0.138
ICH (48)	753.5	0.012
Sepsis (37)	375.3	0.765
COPD (19)	653.4	0.239
Cardiac disease (20)	349.2	0.719
Malignant patients (15)	143	0.197
Post operation (20)	370.7	0.802
**Medical comorbidity**		
Cardiovascular disease (194)	366.7	0.488
Chronic lung disease (59)	447.1	0.803
ESRD (25)	237.5	0.284
Neurologic disease (95)	359.6	0.534
Metabolic disease (113)	398.3	0.829
Malignant diseases (49)	176.2	0.045
Liver disease (25)	202.2	0.195
**Number of comorbidities**		
None (22)	1127.2	< 0.001
One (76)	368.3	0.641
Two (95)	367.9	0.598
Three (70)	425.8	0.941
Four or more (33)	185.9	0.114

*The *P*-values are clinical variables of PMV patients versus all 296 PMV patients. NA, not applicable; Y/O, years old; PMV, prolonged mechanical ventilation; ICH, intracranial haemorrhage; COPD, chronic obstructive pulmonary disease; ESRD, End-Stage Renal Disease.

With regard to the mortality rate of PMV patients (Table 2), the 3-month mortality rate, 6-month mortality rate, 1-year mortality rate, 3-year mortality rate, and 5-year mortality rate among the following patient categories varied statistically significantly: (1) Successfully weaned patients and unsuccessfully weaned patients; (2) Receipt or non-receipt of tracheostomy patients; (3) Aged < 75 years and aged ≥ 75 years; (4) PMV patients among the number of comorbidities. There were only statistically significant differences in the 3-year mortality rate and 5-year mortality rate among patients in the causes of respiratory failure led to PMV (main related to ICH patients). No differences in the mortality rate were found among PMV patients with different medical comorbidities. Discharged PMV patients had the best 1-year mortality rate, 3-year mortality rate, 5-year mortality rate, and mean survival time, as measured by their discharge status in relation to the mortality rate (Table 3). According to age cohorts, the 1-year mortality rate was 50% in the youngest cohort (< 45 years) and 92.1% in that oldest cohort (≥ 85 years) (*P* <0.001). The 3-year mortality rate was 66.7% in the youngest cohort (< 45) years and 100% in the oldest cohort (≥ 85 years) (*P* < 0.001). The 5-year mortality rate was 66.7% in the youngest cohort (< 45 years) and 100% the oldest cohort (≥ 85 years) (*P* < 0.001). Ages greater than 75 exhibited significantly shorter mean survival times (Table 4).

**TABLE 2:** The mortality rates of prolonged mechanical ventilation patients.

Variable, (patient number)	3 Ms	6 Ms	1 Y	3 Ys	5 Ys
All patients (296)	62.50%	72.00%	79.70%	85.50%	89.20%
Successful weaning (179)	49.20%	59.80%	71.50%	79.90%	85.50%
Unsuccessful weaning (117)	82.90%	90.60%	92.30%	94.00%	94.90%
Tracheostomy (45)	40.00%	48.90%	57.80%	73.30%	77.80%
Non-tracheostomy (251)	66.50%	76.10%	83.70%	87.60%	91.60%
Ventilator dependent (42)	47.60%	71.40%	78.60%	85.70%	85.70%
Age < 75 Y/O (131)	52.70%	62.60%	70.20%	77.10%	80.20%
Age ≥ 75 Y/O (165)	70.30%	79.40%	87.30%	92.70%	96.40%
**Causes of respiratory failure led to PMV**					
Pneumonia (104)	59.60%	71.20%	82.70%	91.30%	96.20%
ICH (48)	54.20%	56.30%	64.60%	70.80%	75.00%
Sepsis (37)	75.60%	81.10%	81.10%	86.50%	91.90%
COPD (19)	63.20%	73.70%	73.70%	78.90%	78.90%
Cardiac disease (20)	75.00%	90.00%	90.00%	90.00%	90.00%
Malignant patients (15)	73.30%	80.00%	93.30%	93.30%	100%
Post operation (20)	75.00%	85.00%	90.00%	90.00%	90.00%
**Medical comorbidity**					
Cardiovascular disease (194)	63.90%	74.20%	82.00%	88.70%	91.80%
Chronic lung disease (59)	57.60%	71.20%	74.60%	88.10%	91.50%
ESRD (25)	76.00%	84.00%	92.00%	92.00%	92.00%
Neurologic disease (95)	61.10%	74.70%	81.10%	88.40%	91.60%
Metabolic disease (113)	67.30%	72.60%	81.40%	86.70%	90.30%
Malignant diseases (49)	77.60%	79.60%	89.80%	93.90%	98.00%
Liver disease, (25)	64.00%	76.00%	88.00%	96.00%	96.00%
**Number of comorbidity**					
None (22)	36.40%	40.90%	50.00%	50.00%	59.10%
One (76)	67.10%	77.60%	84.20%	88.20%	92.10%
Two (95)	60.10%	70.50%	78.90%	89.50%	91.60%
Three (70)	65.70%	71.40%	78.60%	84.40%	90.00%
Four or more (33)	69.70%	84.80%	93.90%	97.00%	97.00%

Ms, months; Y, year; Ys, years; Y/O, years old; PMV, prolonged mechanical ventilation; ICH, intracranial haemorrhage; COPD, chronic obstructive pulmonary disease; ESRD, End-Stage Renal Disease.

**TABLE 3:** Characteristics related to discharge status of prolonged mechanical ventilation patients.

	Total patients	Successful weaning patients	Discharged PMV patients*	Ward mortality patients^%^	Ventilator dependent patients	RCC mortality patients^#^
Patients, No	296	179	103	76	42	75
LOS of RCC (days)	20.7	20.8	20.6	21.0	29	15.8
duration of invasive ventilation days)	17.7	15.8	15.6	16.0	29	15.8
Tracheostomy, No	45 (15.2%)	24 (13.4%)	19 (18.4%)	5 (6.6%)	12 (28.6%)	8 (10.7%)
One-year mortality rate	79.7%	71.5%	50.5%	100%	78.6%	100%
Three-year mortality rate	85.5%	79.9%	64.1%	100&	85.7%	100%
Five-year mortality rate	89.2%	85.5%	74.7%	100%	85.7%	100%
Mean survival time (days)						
	417.9	578.4	1003.2	42.3	451.8	15.8

PMV, prolonged mechanical ventilation; RCC, respiratory care center; LOS, length of stay; No, number.

*Discharged PMV patient was defined as successfully weaned PMV patient who was discharged from the hospital.

^%^Ward mortality patient was defined as successfully weaned PMV patient who died in the hospital before discharge.

^#^RCC mortality patient was defined as patient who died in the RCC.

**TABLE 4:** The clinical characteristics and survival outcomes of prolonged mechanical ventilation patients (according to age cohorts).

	< 45 Y/O, No	45–54 Y/O, No	55–64 Y/O, No	65–74 Y/O, No	75–84 Y/O, No	≥ 85 Y/O, No	P-value*
Patients, No	6	29	33	63	114	51	
Successfully wean, No	5 (83.3%)	15 (51.7%)	20 (60.6%)	46 (73.0%)	62 (54.3%)	31 (60.7%)	0.610
Tracheostomy, No	2 (33.3%)	5 (17.2%)	7 (21.2%)	16 (25.3%)	11 (9.6%)	4 (7.8%)	0.011
Mortality rate < 1 year, No	3 (50.0%)	20 (68.9%)	20 (60.6%)	49 (77.7%)	97 (85.1%)	47 (92.1%)	< 0.001
Mortality rate < 3 years, No	4 (66.7%)	22 (75.8%)	22 (66.7%)	53 (84.1%)	101 (88.6%)	51 (100%)	< 0.001
Mortality rate < 5 years, No	4 (66.7%)	23 (79.3%)	25 (75.5%)	54 (85.7%)	108 (94.7%)	51 (100%)	< 0.001
Mean survival time (days)	998.5	682.5	821.0	491.7	297.4	116.3	

*The trend in change of patient number analyzed by linear-by-linear association in chi-square test. No, number; Y/O, years old.

Univariate analysis of 5-year survival rates revealed statistically significant differences between patients who survived < 5 years and those who survived ≥ 5 years with regard to age ≥ 75 years, successful weaning, no comorbidities, receipt or non-receipt of tracheostomy, pneumonia patients and ICH patients (Table 5). Multivariate analysis revealed that those with successful weaning, those with no comorbidities, and those who received tracheostomy had better 5-year survival rates, whereas those aged ≥ 75 years had poorer 5-year survival rates (Table 6).

**TABLE 5:** Comparison of five year survival rate with clinical variables of prolonged mechanical ventilation patients (*N* = 296).

Variable, (patient number)	Survival <5 year (*n* = 265)^a^	Survival ≥5 year (*n* = 31)^a^	*P*	OR
Successful weaning (179)	153 (57.7%)	26 (83.9%)	0.008	3.807
Tracheostomy (45)	35 (13.2%)	10 (32.3%)	0.007	3.129
Ventilator dependent (42)	36 (13.6%)	6 (19.4%)	0.387	1.527
Age more than 75 years (165)	159 (60.0%)	6 (19.4%)	*P* < 0.001	0.160
**Causes of respiratory failure led to PMV**				
Pneumonia (104)	100 (37.7%)	4 (12.9%)	0.011	0.244
Intracranial hemorrhage (48)	36 (13.6%)	12 (38.7%)	0.001	4.018
Sepsis (37)	34 (12.8%)	3 (9.7%)	0.617	0.728
COPD (19)	15 (5.7%)	4 (12.9%)	0.131	2.461
Cardiac disease (20)	18 (6.8%)	2 (6.5%)	0.943	0.946
Malignant patients (15)	15 (5.7%)	0 (0%)	0.377	1.110
Post operation (20)	18 (6.8%)	2 (6.5%)	0.943	0.946
**Medical comorbidity**				
Cardiovascular disease (194)	178 (67.2%)	16 (51.6%)	0.089	0.521
Chronic lung disease (59)	54 (20.4%)	5 (16.1%)	0.576	0.751
ESRD (25)	23 (8.7%)	2 (6.5%)	0.674	0.726
Neurologic disease (95)	87 (32.8%)	8 (25.8%)	0.430	0.712
Chronic liver disease (25)	24 (9.1%)	1 (3.2%)	0.292	0.335
Metabolic disease (113)	102 (38.5%)	11 (35.5%)	0.745	0.879
Malignant diseases (49)	48 (56.7%)	1 (3.2%)	0.066	0.151
**Number of comorbidities**				
None (22)	13 (4.9%)	9 (29.0%)	*P* < 0.001	7.930
One (76)	70 (26.4%)	6 (19.4%)	0.397	1.496
Two (95)	87 (32.8%)	8 (25.8%)	0.430	1.405
Three (70)	63 (23.8%)	7 (22.6%)	0.882	1.069
Four or more (33)	32 (12.1%)	1 (3.2%)	0.171	0.935

^a^Data are listed as numbers of patients and percentages.

OR, odds ratio; RCC, respiratory care center.

**TABLE 6:** The difference in clinical variables and tracheostomy between prolonged mechanical ventilation (PMV) patients who survived < 5 year and those who survived ≥ 5 years.

	odds ratios	95% confidence	*P*
**Univariate analysis**			
Age ≥ 75 years	0.16	0.063–0.403	< 0.001
Successfully weaned	3.807	1.418–10.220	0.008
Zero comorbidities	7.93	3.051–20.609	< 0.001
Tracheostomy	3.129	1.361–7.196	0.007
Pneumonia	0.244	0.083–0.719	0.011
ICH	4.018	1.799–8.974	0.001
Multivariate analysis*			
Age ≥ 75 years	0.227	0.082–0.630	0.004
Successfully weaned	3.471	1.165–10.336	0.025
Zero comorbidities	8.45	2.691–26.537	< 0.001
Tracheostomy	3.626	1.367–9.617	0.01

*The factors showed the *P*-value < 0.05 of univariate analysis and entered the multivariate analysis. PMV, prolonged mechanical ventilation; ICH, intracranial haemorrhage.

**FIGURE 2:**
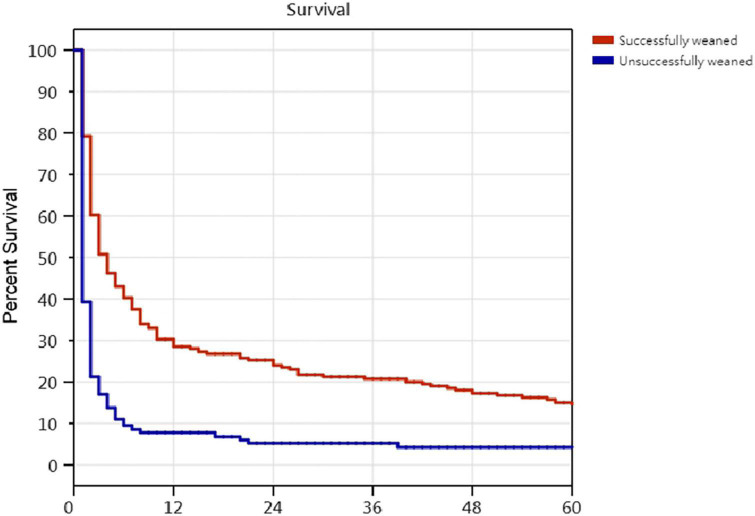
The Kaplan–Meier curves of successfully weaned and unsuccessfully weaned prolonged mechanical ventilation patients. Cox proportional hazards regression analyses of 296 prolonged mechanical ventilation patients, successful weaning were correlated with a reduction in the risk of death by 51.6% [*P* = 0,001; hazard ratio (HR) = 0.484; 95% CI 0.376–0.624].

Kaplan–Meier survival curves for successfully weaned patients versus unsuccessfully weaned patients, patients who received or did not receive tracheostomy, patients aged < 75 years versus patients aged ≥ 75 years, PMV patients among the causes of respiratory failure led to PMV, PMV patients among different medical comorbidities, and PMV patients among the number of comorbidities are illustrated in Figure 2 to Figure 7. These 296 PMV patients underwent Cox proportional hazards regression analysis. The results are as follows: (1) Successful weaning was correlated with a reduction in the risk of death by 51.6%. (2) Receiving a tracheostomy was correlated with a 41.2% reduction in the risk of death. (3) Patients less than 75 years were correlated with a reduction in the risk of death by 37.9%. (4) Correlation with ICH was correlated with a reduction in the risk of death by 37.6%. (5) Patients without comorbidities were correlated with a reduction in the risk of death by 60.5%.

**FIGURE 3:**
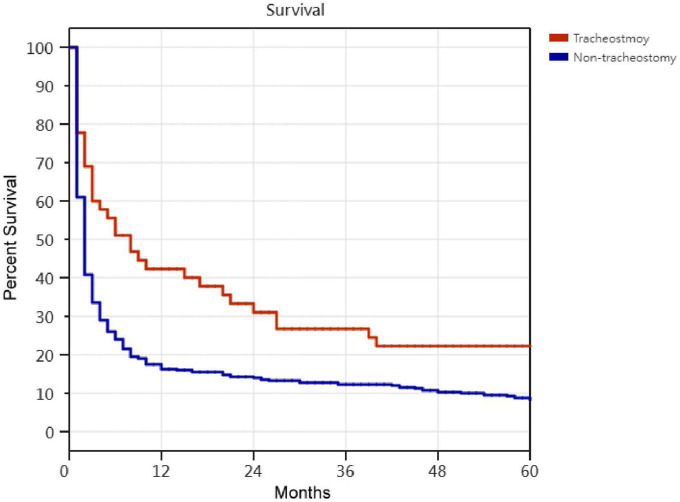
The Kaplan–Meier curves of receipt and non-receipt of tracheostomy PMV patients. Cox proportional hazards regression analyses of 296 prolonged mechanical ventilation patients, receipt of tracheostomy were correlated with a reduction in the risk of death by 41.2% [*P* = 0.001; hazard ratio (HR) = 0.588; 95% CI 0.411–0.841].

**FIGURE 4:**
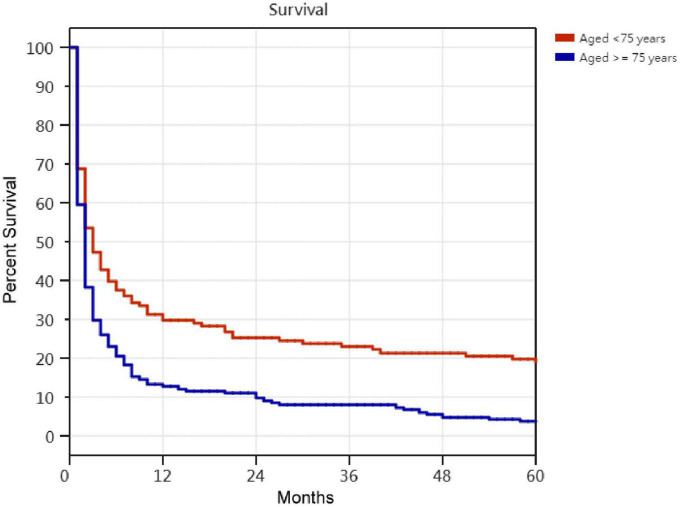
The Kaplan–Meier curves of aged <75 years and aged ≥75 years prolonged mechanical ventilation patients. Cox proportional hazards regression analyses of 296 prolonged mechanical ventilation patients, in patients aged <75 years were correlated with a reduction in the risk of death by 37.9% [*P* < 0.001; hazard ratio (HR) = 0.621; 95% CI 0.484–0.797].

**FIGURE 5:**
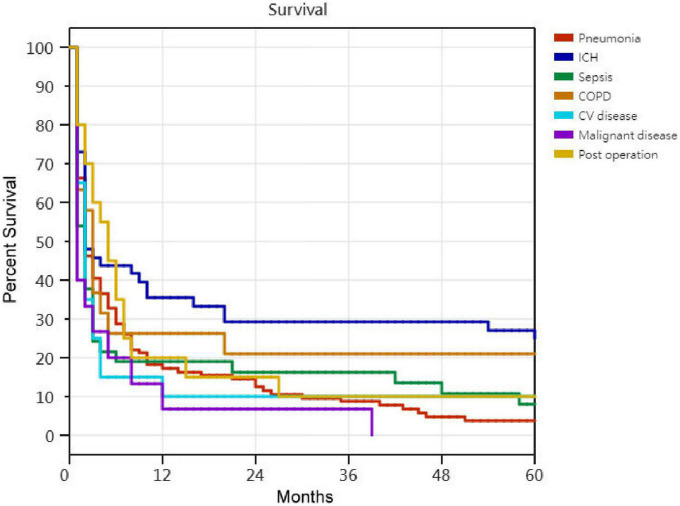
The Kaplan–Meier curves of patients among the causes of respiratory failure led to prolonged mechanical ventilation. Cox proportional hazards regression analyses of 296 prolonged mechanical ventilation patients, in ICH patients were correlated with a reduction in the risk of death by 37.6% [*P* = 0.003; hazard ratio (HR) = 0.624; 95% CI 0.438–0.889].

**FIGURE 6:**
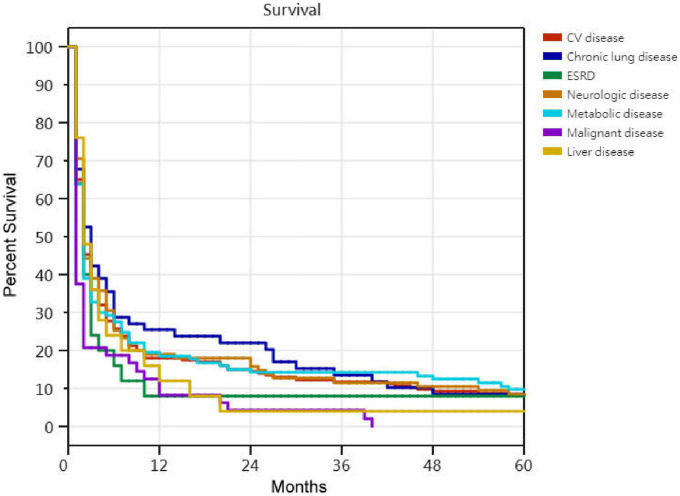
The Kaplan–Meier curves of prolonged mechanical ventilation patients among different medical comorbidities. No statistically significant differences in Kaplan–Meier curves of prolonged mechanical ventilation patients among different medical comorbidities.

**FIGURE 7:**
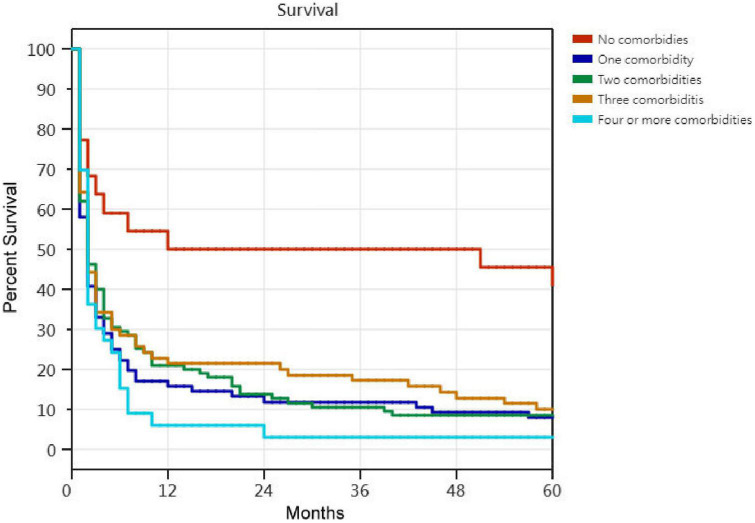
The Kaplan–Meier curves of prolonged mechanical ventilation patients among the number of comorbidities. Cox proportional hazards regression analyses of 296 prolonged mechanical ventilation patients, in patients without comorbidities were correlated with a reduction in the risk of death by 60.5% [*P* < 0.001; hazard ratio (HR) = 0.395; 95% CI 0.225–0.693].

There were only twenty patients alive until Dec 31, 2011. Among the 276 dead patients, the causes of death included 133 from pneumonia, 46 from sepsis, 29 from sudden death, 18 from malignant diseases, 12 from cardiac diseases, 12 from COPD, 8 from respiratory failure, and 18 from miscellaneous causes. The number of mortality patients was distributed among 185 patients at 3 months, 28 patients at 6 months, 23 patients at 1 year, 17 patients at 3 years, 12 patients at 5 years and 11 patients at more than 5 years. During the 10-year period, patient mortality decreased from 185 patients (62.5%) at 3 months to 11 patients (3.7%) at more than 5 years (*P* = 0.001, linear-by-linear association chi-squared test). Pneumonia in 133 PMV patients (48.2%) was the leading cause of death in PMV patients, including 99 patients with detected pneumonia pathogens (Table 7). Overall, 53 of the detected pneumonia pathogens were polymicrobial infections. The most frequent pneumonia pathogens in PMV patients were *Acinetobacter baumannii* (48 cases, 48.5%), *Pseudomonas aeruginosa* (40 cases, 40.4%), and *Klebsiella pneumoniae* (16 cases, 16.2%). A total 59.6% (59 cases) of the detected pneumonia pathogens were carbapenem-resistant Gram-negative bacilli, with carbapenem-resistant Acinetobacter baumannii (CRAB) accounting for 43.4% of those occurrences (43 cases).

**TABLE 7:** Pneumonia pathogens of prolonged mechanical ventilation (PMV) death patients.

Pathogens	Patients number (*N* = 133)
No-detected pneumonia pathogen	43
Detected pneumonia pathogen	99
CRAB	43
CRPA	8
CRKP	8
Acinetobacter baumannii	5
Pseudomonas aeruginosa	32
Klebsiella pneumonia	8
Burkholderia cepacia	11
Stenotrophomonas maltophilia	7
Escherichia coli	9
Enterobacter cloacae	2
Proteus mirabilis	4
Serratia marcescens	1
Hemophilus infuenza	3
Morganella morganii	3
Providencia stuartii	3
Ralstonia	2
MRSA	9
MSSA	3
MRSE	1
Alcaligenes faecalis	1

PMV, prolonged mechanical ventilation; N, number; CRAB, carbapenem-resistant Acinetobacter baumanni; CRPA, carbapenem-resistan Pseudomonas aeruginosa; CRKP, carbapenem-resistan Klebsiella pneumonia; MRSA, Methicillin-resistant Staphylococcus aureus; MSSA, methicillin-susceptible Staphylococcus aureus; MRSE, Methicillin-resistant Staphylococcus epidermidis.

## Discussion

There is no article exploring the survival time of PMV patients. Our study showed that successfully weaned PMV patients, PMV patients undergoing tracheostomy, patients aged less than 75 years, ICH patients, and patients without comorbidities had better survival times. In addition, the 5-year mortality rates of patients receiving PMV have not been investigated extensively. The pooled mortality between 2 and 4 years among acute care hospital weaning units was 56% (range 4–66%) (7). Stoller et al., showed that the 1-year mortality rate, 3-year mortality rate, and 5-year mortality rates for162 PMV patients were 57, 73, and 81%, respectively. Younger age was significantly associated with longer survival (2). Our series showed worse 1-year mortality rates, 3-year mortality rates, and 5-year mortality rates than the series in the literature. Our study showed that patients who were successfully weaned from PMV, who received a tracheostomy, were less than 75 years old and had no comorbidities had a lower 1-year mortality rate, 3-year mortality rate, and 5-year mortality rate.

The differences between PMV patients who survived more than 5 years and those who survived less than 5 years have not been investigated in the literature. Our research demonstrated that factors such as age under 75, the absence of comorbidities, successful weaning from PMV, and tracheostomy placement contributed to PMV patients who survived more than 5 years. Studies indicate that older age, failure to wean, four or more comorbidities and end-stage renal disease (ESRD) comorbidity were associated with poor 1-year survival rates for PMV patients (2, 8–14). No comorbidities and successful weaning of PMV patients were two factors related to a better 1-year survival rate. The influential factors of 1-year and 5-year survival rates were similar except for tracheostomy.

With regard to tracheostomy, in a study by Engoren et al., patients liberated from mechanical ventilation and discharged from the hospital with a tracheostomy had worse long-term outcomes (15). In a study by Warnke et al., successfully weaned PMV patients with a closed tracheostomy had a higher survival rate than patients with a permanent tracheostomy (16). Successfully weaned from PMV patients and decannulation of tracheostomy after discharge from the hospital means that those patients have a less functional impairment and better pulmonary physiologic status. The physician decided to reverse the tracheostomies for those patients, and those PMV patients should have a better long-term outcome. For ICU patients, the US literature debates early tracheostomy or late tracheostomy, which offers a better treatment plan and a better prognosis for patients on ventilators. According to studies by Huang et al., Taiwanese PMV patients who have undergone tracheostomy have a positive 1-year survival rate, a noticeably lower in-hospital mortality rate, and a lower risk of developing ventilator-associated pneumonia (17). According to research by Wu et al., patients who have tracheostomy had a lower in-hospital mortality rate than those who do not (18). Taiwan’s RCC PMV patients typically have to choose whether they require a tracheostomy rather than an early or late one. In Taiwan, PMV patients are encouraged to undergo a tracheostomy in the weaning unit. The majority of patients and their families, however, are opposed to this surgery. The most frequent justifications for refusing surgery are concern that the procedure will leave a wound on the patient’s neck, worry about the risks and complications of tracheostomy, and inaccurate subjective opinions of the family members. The following are examples of false beliefs regarding tracheostomy: (1) it will prolong the patient’s illness and increase the family’s hardship; (2) It will make the patient’s life shorter and cause more suffering; (3) following a tracheostomy, the tube cannot be removed permanently, leaving the patient bedridden for the rest of their lives. As a result, relatives may think it would be preferable to let the patient to experience adverse effects and suffering following endotracheal tube intubation than to permit tracheostomy (19). According to Taiwan’s Clinical Performance Indicators data, 39% of PMV patients at the RCC medical center underwent tracheostomies. Compared to the US, Taiwan has a lower proportion of PMV patients that undergo require tracheostomy. Our previous study showed that only 37 PMV patients (9.7%) underwent tracheostomy during a 3-year period (19). The clinical situation of PMV patients receiving or not receiving tracheostomy in Taiwan is different from that in Western countries. Most PMV patients underwent tracheostomy because these patients needed permanent tracheostomy after discharge from the hospital. In actuality, a large number of PMV patients require a permanent tracheostomy but do not receive one. Therefore, receiving tracheostomy positively influences the long-term survival outcomes of PMV patients in Taiwan.

Furthermore, when compared to Taiwanese medical centers, our hospital has a low rate of tracheostomy. Medical centers are located in cities such as Taipei, Taichung, Kaohsiung, Tainan. The majority of people have excellent levels of education and living standards in city. Our hospital is located in the remote community. The majority of the population is elderly. In older generations believed that when someone passes away, their body should be free of any wounds. Family members are opposed to the treatment because they do not want their loved ones to have a permanent tracheostomy wound in their necks.

With regard to survival cures, our study showed that successfully weaned from PMV patients, receipt of tracheostomy PMV patients, age less than 75 years old patients, no comorbidities patients and ICH PMV patients had better overall survival outcomes. The survival cure analysis was advanced to confirm the findings of influential factors related to overall survival outcomes in PMV patients.

Pneumonia was the leading cause of death in our PMV patients. Carbapenem-resistant gram-negative bacilli account for 59.6% of known pneumonia pathogens, especially Carbapenem-resistant *Acinetobacter baumannii*. In a study by Chien, prior exposure to piperacillin/tazobactam and imipenem was found to be strongly linked with the rise in multidrug-resistant Acinetobacter baumannii. In patients receiving PMV, multidrug-resistant microorganism pneumonia was linked to an elevated 6-month mortality rate (20). Another Taiwanese study found that prior use of imipenem, meropenem, piperacillin/tazobactam, or fourth-generation cephalosporins was an independent risk factor for extensive drug-resistant Acinetobacter baumannii infections in hospitals (21). For PMV patients, carbapenem-resistant gram-negative bacilli infection is a potentially lethal problem, so we must utilize broad-spectrum antibiotics wisely, notably the stringent usage of carbapenems in our hospital.

### Limitation

The long-term survival outcomes of PMV patients were the focus of this study. Acute Physiology and Chronic Health Evaluation II scores, laboratory data, respiratory measurements, or any other similarly pertinent characteristics were not gathered from the patients. As a result, we were unable to identify which of these metrics, if any, was associated with the long-term survival results of patients who underwent PMV. It is crucial to understand the acute critical stage of PMV patients’ laboratory data and Acute Physiology and Chronic Health Evaluation II scores but less crucial to comprehend the patients’ long-term survival outcomes. The respiratory parameters are connected to a patient’s ability to wean from PMV. Although few, no previous publications have thoroughly covered the long-term mortality rate of PMV patients. Our study’s findings can provide the medical community with advanced long-term survival outcomes for PMV patients. Because this study was retrospective in nature and conducted at a single weaning center, it is important to interpret the findings about the long-term survival outcomes of PMV patients with caution. This may not be applicable to other weaning units. A better understanding of long-term outcomes is needed, and efforts are urgently needed to improve survival. Randomized controlled trials are needed to compensate for the shortcomings associated with a single-center retrospective study.

## Conclusion

The 5-year mortality rates of PMV patients were significantly dependent on successful weaning, receipt of tracheostomy, age less than 75 years and no comorbidities. According to our study, successful weaning was linked to a risk of death reduction of 51.6%, tracheostomy placement was linked to a risk of death reduction of 41.2%, patients younger than 75 years old were linked to a risk of death reduction of 37.9%, and patients with no comorbidities were linked to a risk of death reduction of 60.5%. For PMV patients, carbapenem-resistant gram-negative bacilli infection is a potentially lethal problem, so we must utilize broad-spectrum antibiotics wisely, notably the stringent usage of carbapenems in our hospital. Despite the tragedy of long-term survival outcomes of PMV patients, clinicians should never give up on the dream of improving long-term outcomes.

## Data availability statement

The raw data supporting the conclusions of this article will be made available by the authors, without undue reservation.

## Ethics statement

The studies involving human participants were reviewed and approved by the Buddhist Dalin Tzu Chi Hospital Research Ethics Committee (Approved IRB No. B10802009). Written informed consent for participation was not required for this study in accordance with the national legislation and the institutional requirements.

## Author contributions

The author confirms being the sole contributor of this work and has approved it for publication.
